# Spindle Cell Malignancy and Asbestos Exposure: A Case Report

**DOI:** 10.7759/cureus.54382

**Published:** 2024-02-17

**Authors:** Nakoma Walker, Chinedum Okafor, Nitesh Gandhi, Shivani Sharma

**Affiliations:** 1 Internal Medicine, Louisiana State University Health Sciences Center, Shreveport, USA; 2 Pathology, Louisiana State University Health Sciences Center, Shreveport, USA

**Keywords:** screening, asbestos, incidental finding, lung cancer, pleural effusion, sarcoma, spindle cell, case report

## Abstract

We outline the presentation of a 68-year-old woman who received a chest radiograph due to her insurance requirements, resulting in the discovery of a left-sided pleural effusion. The effusion was further characterized as loculated on subsequent imaging. Thoracentesis yielded exudative fluid, leading to the patient undergoing video-assisted thoracoscopic surgery (VATS). During this procedure, a cystic mass was visualized, with the conversion of the operation to an open thoracotomy and left lower lobe lobectomy. Pathology was positive for spindle cell sarcoma. A thorough history of the patient revealed a decades-long occupational exposure to asbestos. The significance of this report is to illustrate the clinical presentation, immunohistochemical characteristics, and management of a rare spindle cell malignancy. Our case also raises the importance of screening patients on an individualized, shared decision-making basis.

## Introduction

This article was previously presented as a poster at the Annual Internal Medicine Research Day at Louisiana State University Health Sciences Center on April 13, 2023. Sarcomatoid cancers are known to be rare in nature as well as associated with poorer outcomes [1]. As this type of tumor is more rare in nature, it is key to add to the literature details on associated histology, risk factors, and disease course.

## Case presentation

**Figure 1:**
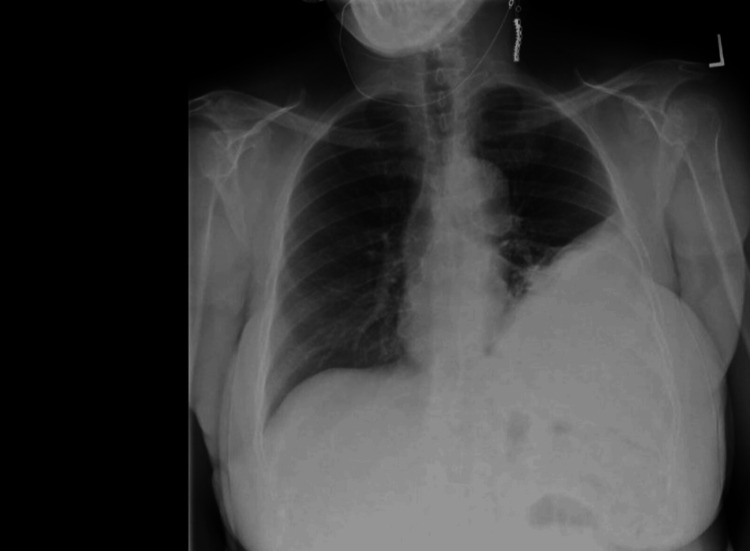
Initial Screening Chest Radiograph Showing Left-Sided Pleural Effusion

**Figure 2:**
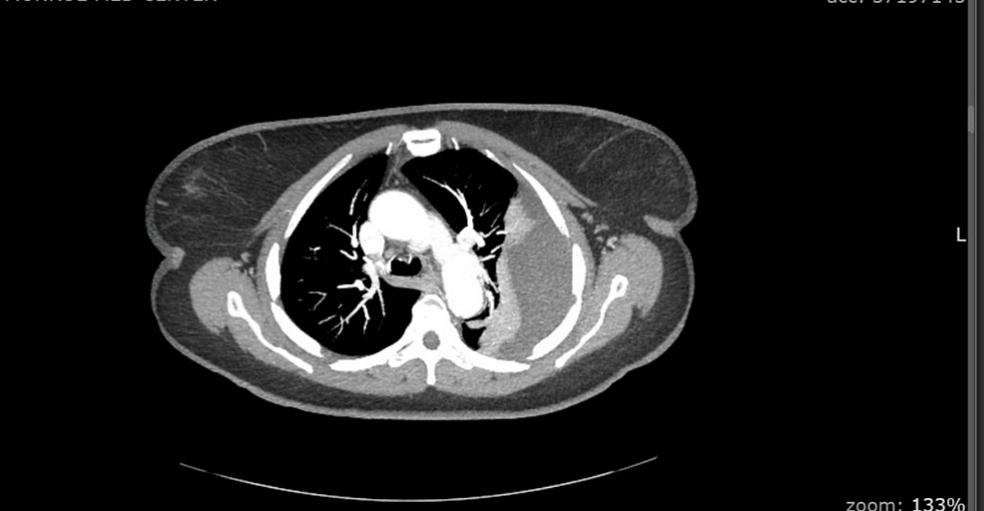
Axial Chest CT With Contrast Showing Large Left-Sided Loculated Pleural Effusion CT, computed tomography

A 68-year-old woman presented to her primary care physician for a screening chest radiograph to fulfill her insurance requirements. At this time a left-sided pleural effusion was found (Figure 1). Her past medical history included hypertension, hyperlipidemia, and type 2 diabetes, with no smoking history. Of note, she had a history of 30-40 years working in an electrical plant with asbestos exposure. She was then referred to our facility for further work-up. Computed tomography (CT) of the chest further characterized the effusion as large with evidence of loculations (Figure 2). The patient was admitted to the hospital for further work-up and management of the pleural effusion. She denied cough, shortness of breath, hemoptysis, or weight loss. Thoracentesis yielded exudative fluid. Cardiothoracic surgery was consulted and video-assisted thoracoscopic surgery (VATS/decortication was performed, during which a large cystic mass was visualized. She underwent an open thoracotomy during which left lower lung lobectomy and thoracic lymph node dissection was performed.

**Figure 3:**
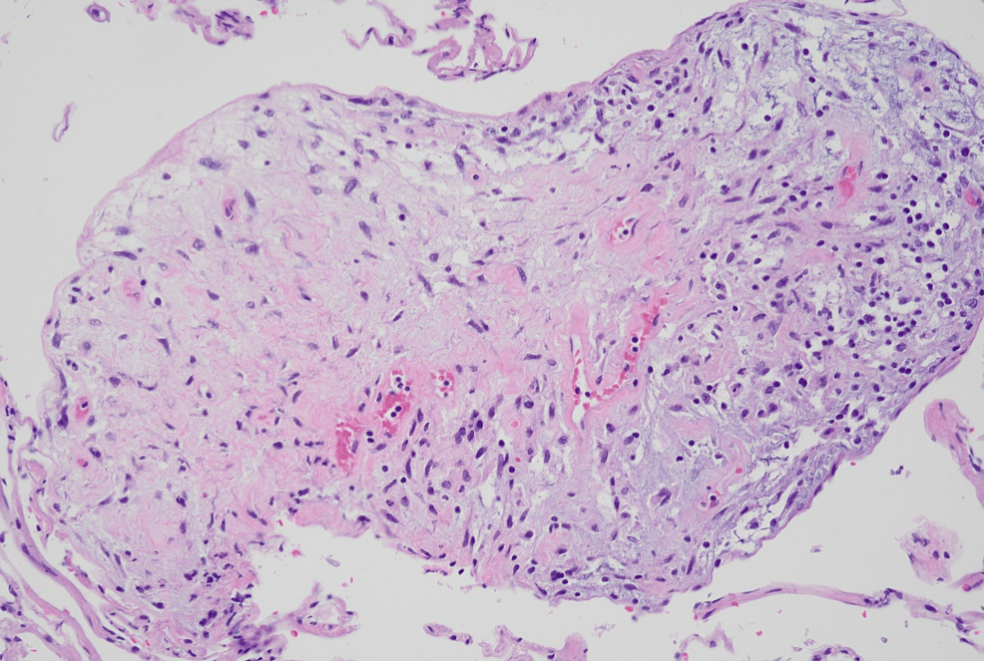
Histology of Spindle Cell Malignancy

**Figure 4:**
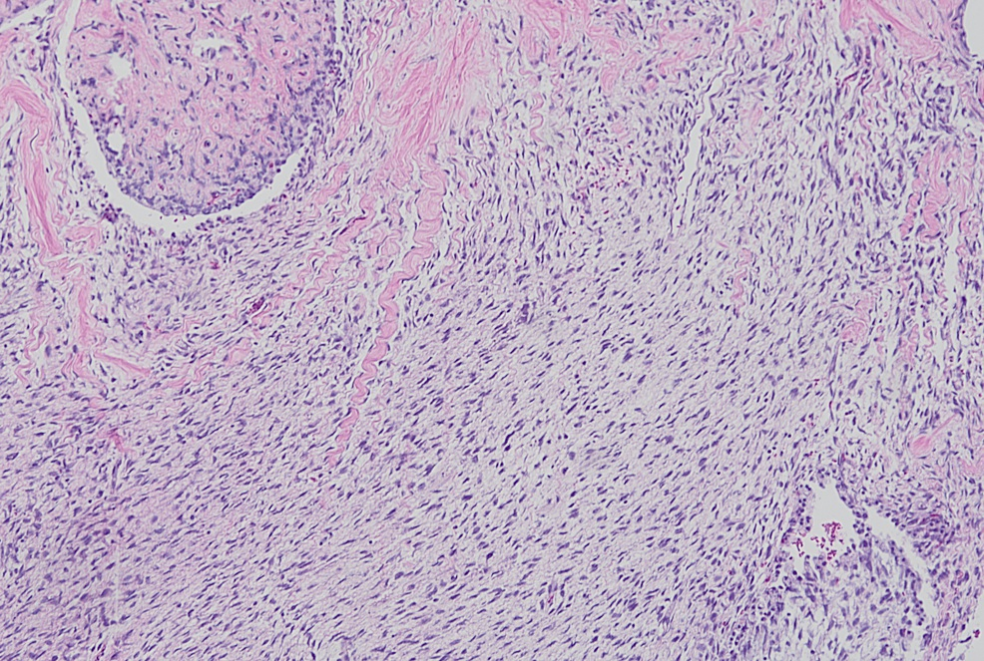
Histology of Spindle Cell Malignancy

**Figure 5:**
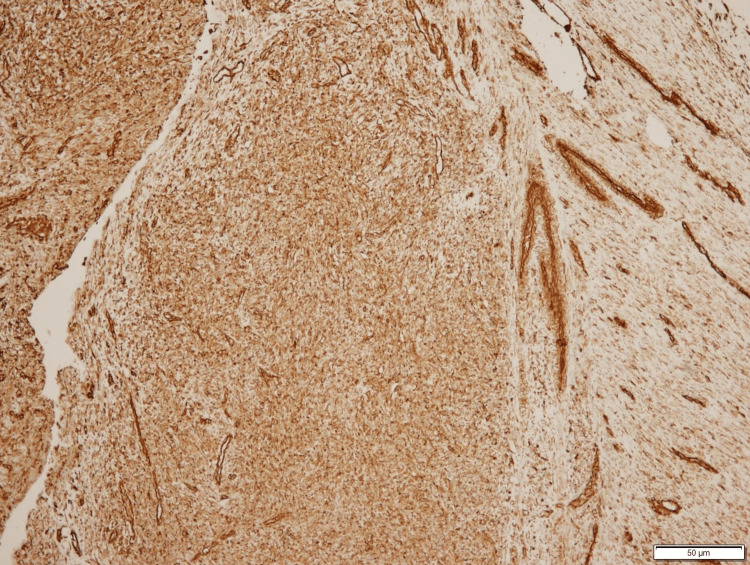
IHC Staining Positive for Vimentin IHC, immunohistochemistry

**Figure 6:**
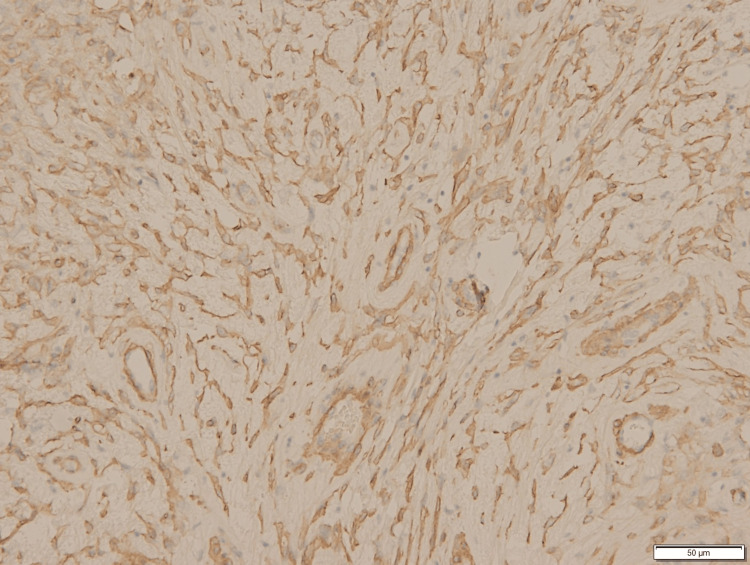
IHC Staining Positive for Smooth Muscle Actin IHC, immunohistochemistry

**Figure 7:**
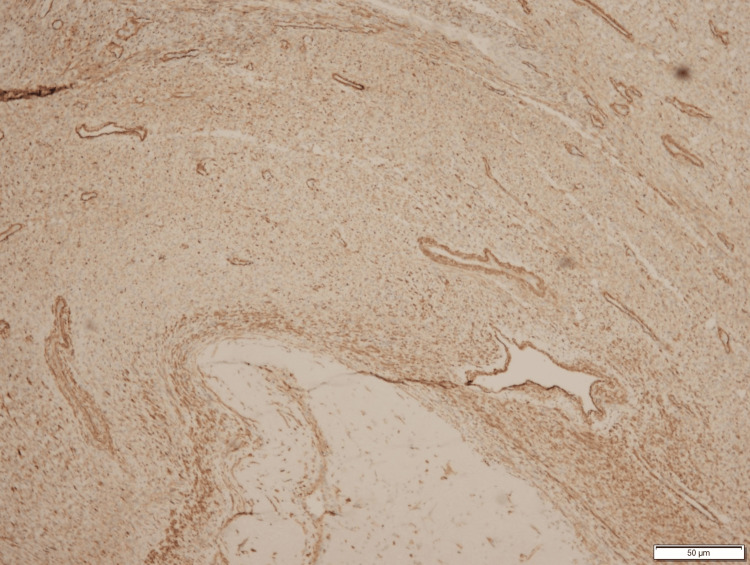
IHC Staining Positive for Caldesmon IHC, immunohistochemistry

**Figure 8:**
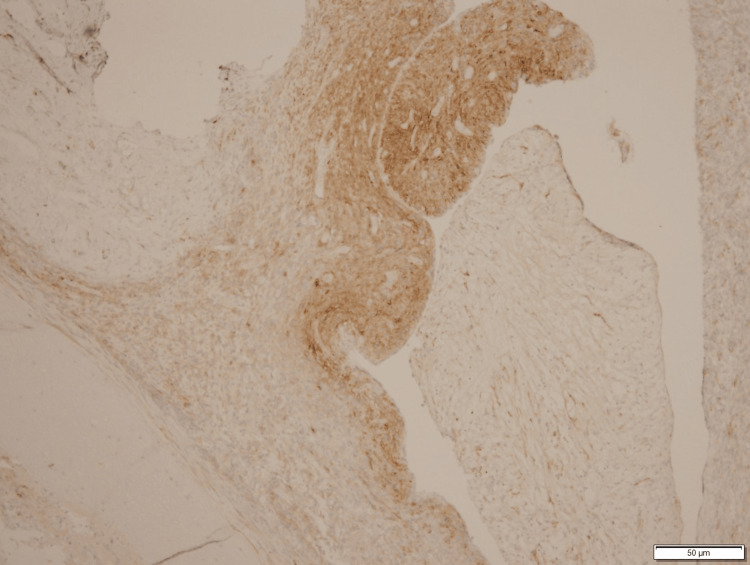
IHC Staining Positive for CD10 IHC, immunohistochemistry

**Figure 9:**
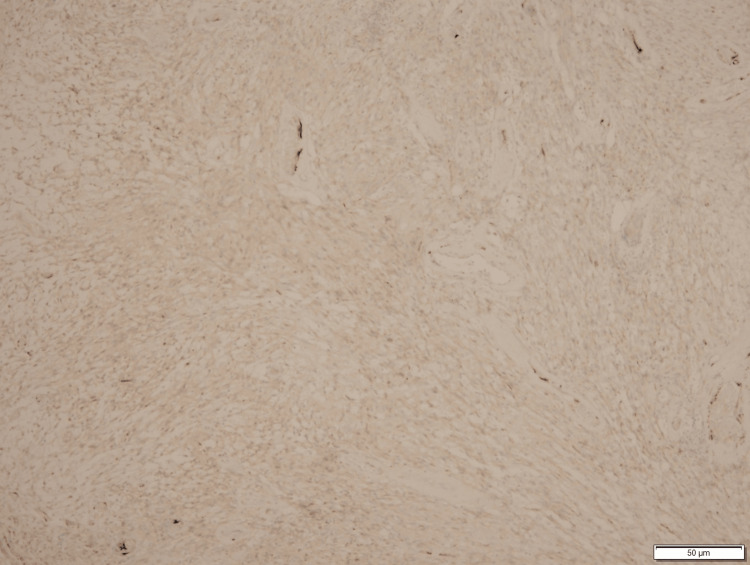
IHC Staining Positive for WT1 (Weak) IHC, immunohistochemistry

**Figure 10:**
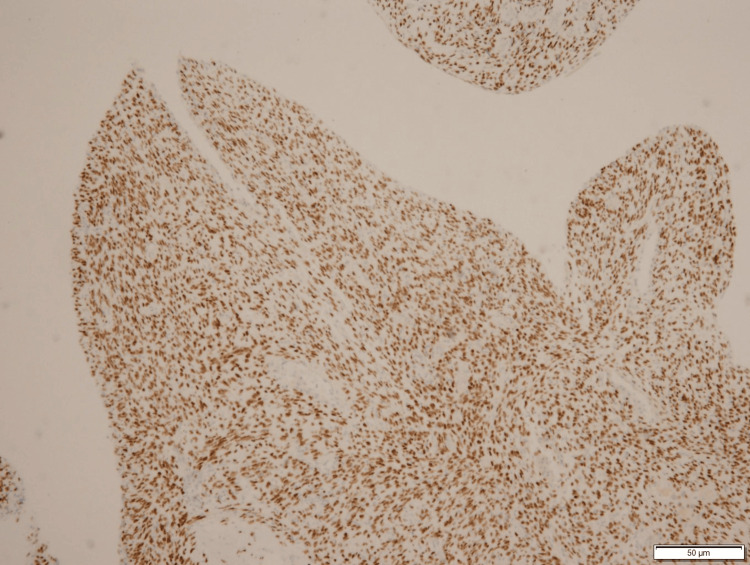
IHC Staining Positive for Estrogen Receptors IHC, immunohistochemistry

**Figure 11:**
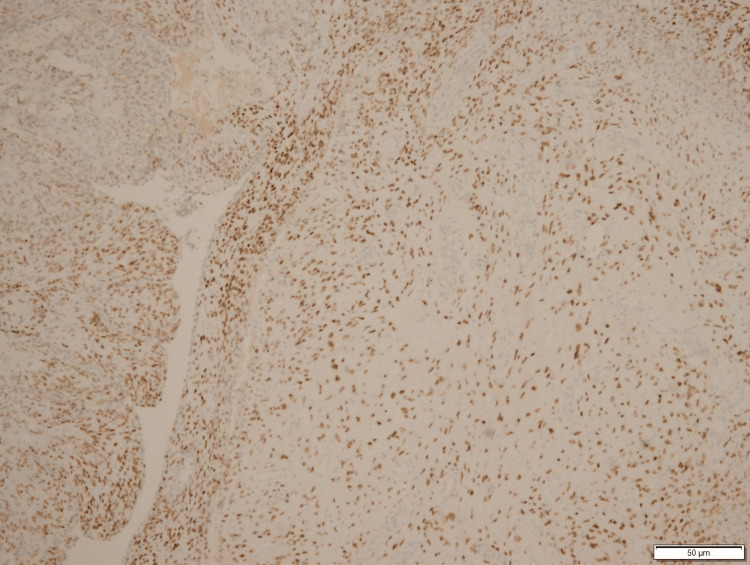
IHC Staining Positive for Progesterone Receptors IHC, immunohistochemistry

Pathology revealed a grade 1 tumor of size 25×13×12.5 cm. Immunohistochemistry (IHC) staining for the spindle cell component was positive for vimentin, smooth muscle actin, caldesmon, CD10, desmin (a subset of tumor cells), WT1 (weak), estrogen, and progesterone receptors. There were no fusion genes present or involvement of lymph nodes (Figures 3-11). Due to the rare nature of the tumor, the final histological interpretation was an estrogen and progesterone receptor-positive mesenchymal tumor with heterologous components (chondromatous). Differentials include sarcoma vs biphasic epithelial and stromal tumors.

**Figure 12:**
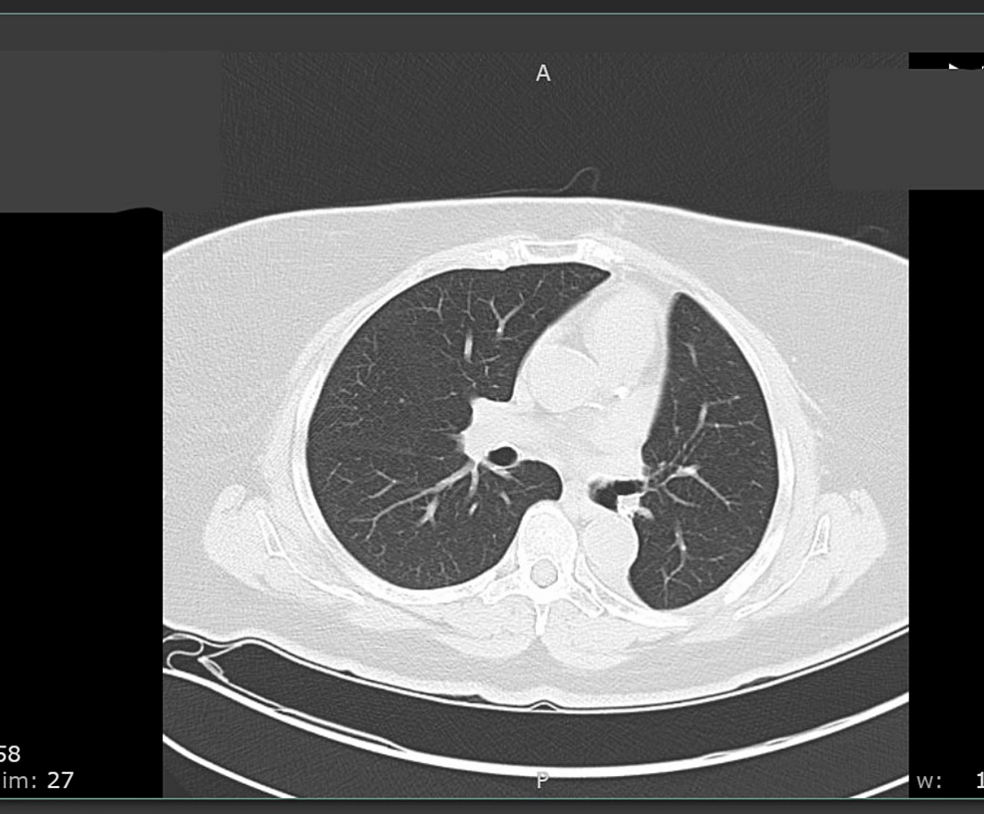
Five Month Post-Operation Axial CT Chest Without Contrast CT, computed tomography

Informed decision-making was engaged with the patient who decided against chemotherapy. Additionally, radiation therapy was decided against as the lung expanded to the site of the previous tumor and the location of the lesion was pleural, not involving the parietal pleura. Positron emission tomography (PET) scan was negative for residual disease. CT of the chest five months post-operation showed resolution of the left pleural effusion, scarring of the left lung base, and no evidence of intrathoracic metastasis (Figure 12). The patient has been adhering to follow-up imaging and clinic appointments with hematology/oncology, pulmonology, and ENT, which has thus far not yielded concern for recurrence of disease or deterioration in clinical status.

## Discussion

A few differentials were provided for our patient's diagnosis, due to the rare nature of her patient's tumor, with sarcoma leading the list. Sarcomatoid cancer is a rare cause of lung malignancy, with the higher end of incidence at approximately 0.4% [1]. Histology often shows a high-grade tumor by the time of diagnosis, with even localized sites of disease associated with a prognosis of less than three years (median 34 months) [1]. Additionally, even when compared with well-matched cohorts of patients with other forms of non-small cell lung cancer, individuals with sarcomatoid cancer had worse overall survival [1]. This malignancy is known to be associated with smoking, is more predominant in men with a ratio of approximately three to one, a median age of 62, and has a common upper lobe peripheral location [2-4].

Our patient had a significant 30-40-year history of occupational asbestos exposure from working in an electrical plant. A systematic review and meta-analysis revealed that 1.1% of asymptomatic workers with asbestos exposure had lung cancer on screening with low-dose CT. For comparison, this is similar to the 1% prevalence of lung cancer in screening heavy smokers [5]. Importantly, approximately 40% of those lung cancers identified were stage one and, therefore, more amenable to removal via surgery. Among the cohort studies included in this analysis, the highest percentage of women represented was approximately 5% [5]. The lack of women included in studies pertaining to asbestos exposure and lung carcinoma is a common theme. In our patient, a screening radiograph led to the discovery of a lung cancer likely linked to her occupational exposure.

One of the included studies in this meta-analysis evaluated asymptomatic patients with asbestos exposure for lung nodules as well as atypical plaques, which could be indicative of other asbestos-related lung pathologies such as mesothelioma [6]. Based on screening guidelines, those suspicious nodules were followed up with more frequent screening and/or biopsy as indicated [6]. The screening modality used was low-dose CT. In those whose initial scans were negative, they were offered an annual low-dose CT scan. The rate of malignancy discovered was 2.1%. An additional study also followed asbestos-exposed individuals with spiral CT and chest radiography. They similarly found lung malignancies via screening with CT. They mention a patient who refused further follow-up after a fine-needle aspiration biopsy yielded an inadequate sample [7]. This highlights the importance of discussions, which must be had with patients, disclosing the further work-up that will be indicated if something is found as well as the possibility of false positive or negative findings. A thorough discussion parsing out the associated possible risk(s) and benefit(s) of such screening described above should be had with all eligible patients.

Finally, the type of modality used for screening is to be considered as well. A 2009 study evaluated a cohort of workers exposed to asbestos for a two-year follow-up period. They were screened with low-radiation helical chest CT scan and chest radiography [8]. Twenty-four lung malignancies were discovered. The sensitivity of the CT scan was higher (at 83% compared to 33% for chest radiographs). However, the chest radiography had a higher specificity (at 95% compared to 78% for CT ). Based on these results, the suggestion arose that CT be used as a screening modality, with an increased threshold for a suspicious non-calcified lung lesion in patients of this population to be placed at 5 mm [8].

## Conclusions

We report the case of a patient whose diagnosis of lung malignancy began with incidental chest radiograph findings. Further imaging revealed a large loculated effusion. Thoracentesis and ultimately VATS were performed where a cystic mass was located and removed via left lower lobe lobectomy. Pathology results provided the diagnosis of spindle cell malignancy. Surgical removal and observation were the therapeutic interventions of choice based on informed decision-making between medical team members and the patient. She follows closely with hematology/oncology and pulmonology, with thus far no evidence of recurrence.

From an asymptomatic presentation to a spindle cell malignancy diagnosis, our case report highlights the value of taking thorough occupational and environmental histories to establish connections such as this. Our final aim is to encourage individualized plans of care (including screening imaging) for patients with a history of asbestos exposure.
